# Supplementary material for: The Role of luxS in Campylobacter jejuni Beyond Intercellular Signaling
Source: Microbiol Spectr. 2023 Feb 1;11(2):e02572-22. doi: 10.1128/spectrum.02572-22 (PMC10100756; doi:10.1128/spectrum.02572-22)
Supplement: Supplemental file 1 — Supplemental material. Download spectrum.02572-22-s0001.pdf, PDF file, 0.6 MB [file spectrum.02572-22-s0001.pdf]

Supplementary Material

SUPPLEMENTARY FIGURES

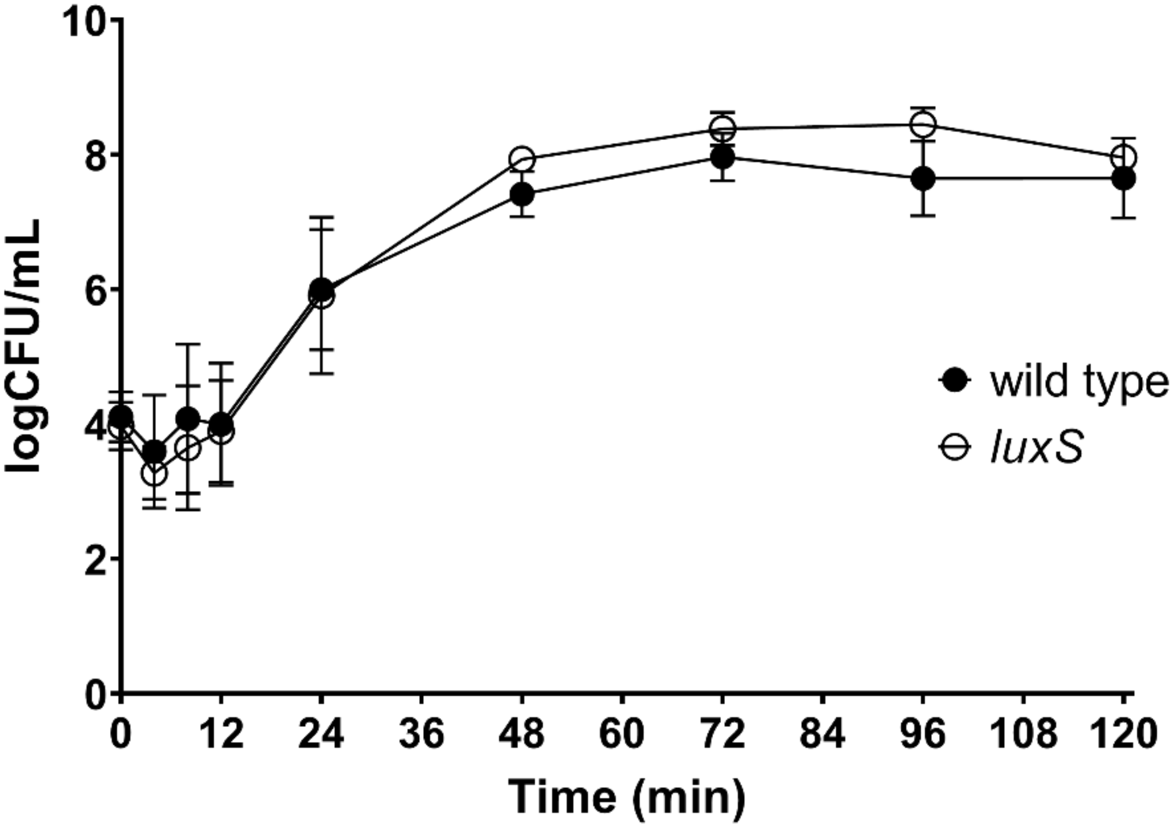

Supplementary Figure S1. Growth of *luxS* mutant and wild type in MH broth at 42°C in microaerobic atmosphere, presented as log_{10}CFU/mL. Data are means ± standard deviation from three replicas. No significant differences were seen versus the relevant controls (Students' t-test).

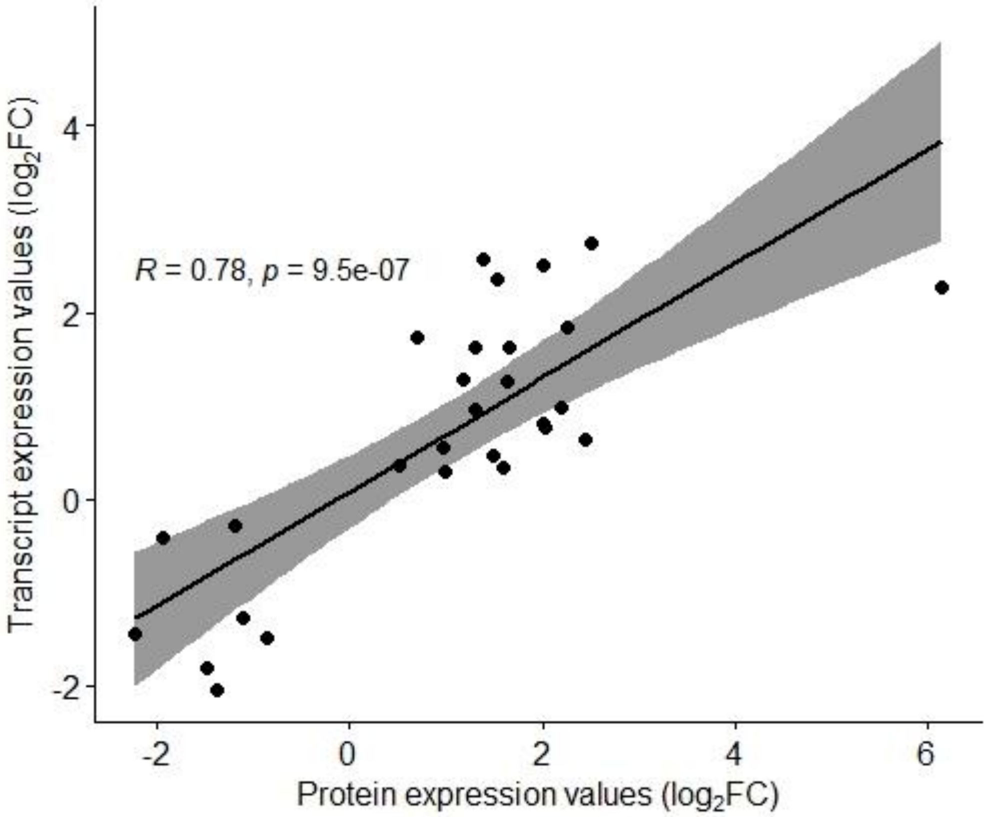

Supplementary Figure S2. Correlation analysis using Pearson's method was performed for the data on transcript and protein expression, which are significantly differentially expressed in both transcriptomic and proteomic datasets and show the same direction of expression levels (n=28). The *P*-value of the test is 9.518e-07, which is below the significance level of alpha = 0.05. It can be concluded that proteomic and transcriptomic expression are significantly correlated with a Pearson's correlation coefficient (*R*) of 0.78 and a *P*-value of 9.518e-07 (t = 6.3711, df = 26).

SUPPLEMENTARY TABLE 1 Differentially expressed genes in *C. jejuni* 11168Δ*luxS* normalized to the *C. jejuni* NCTC 11168 (FDR, P ≤ 0.05). Genes with an absolute $\log_2$ fold change ≥ 2.5 are highlighted in grey.

Gene name	Log_2 fold-change	False discovery rate *P*-value
**Down-regulated**		
dnaN	-3.1	0E + 00
rnhB	-1.8	0E + 00
Cj0021c	-1.2	0E + 00
Cj0034c	-2.1	0E + 00
Cj0036	-3.1	0E + 00
Cj0040	-4.5	0E + 00
fliA	-1.6	0E + 00
Cj0073c	-1.5	0E + 00
Cj0092	-2.1	0E + 00
Cj0093	-1.8	0E + 00
atpF	-1.8	0E + 00
atpF	-2.1	0E + 00
atpH	-2.9	0E + 00
exbB3	-2.6	0E + 00
Cj0114	-4.8	0E + 00
Cj0142c	-2.4	0E + 00
Cj0143c	-4.8	0E + 00
Cj0152c	-3.4	0E + 00
cfbpA	-2.2	0E + 00
def	-2.2	0E + 00
tig	-3.4	0E + 00
infC	-1.4	0E + 00
Cj0229	-2.1	0E + 00
Cj0230c	-1.7	0E + 00

Cj0254	-2.0	0E + 00
Cj0262c	-1.7	0E + 00
Cj0268c	-1.3	0E + 00
lpxB	-2.1	0E + 00
Cj0323	-5.0	0E + 00
ndk	-1.6	0E + 00
polA	-1.4	0E + 00
trpE	-1.5	0E + 00
Cj0371	-2.3	0E + 00
Cj0372	-1.7	0E + 00
Cj0374	-2.7	0E + 00
pyk	-2.1	0E + 00
Cj0397c	-2.9	0E + 00
Cj0403	-1.9	0E + 00
Cj0404	-2.3	0E + 00
Cj0406c	-3.6	0E + 00
Cj0420	-1.8	0E + 00
Cj0421c	-3.1	0E + 00
Cj0424	-2.2	0E + 00
Cj0448c	-1.3	0E + 00
Cj0449c	-2.0	0E + 00
Cj0454c	-2.4	0E + 00
Cj0459c	-3.5	0E + 00
nusA	-1.4	0E + 00
Cj0463	-1.6	0E + 00
rplJ	-1.8	0E + 00
clpB	-2.6	0E + 00
purQ	-2.2	0E + 00
htpG	-2.3	0E + 00
fliE	-2.1	0E + 00
flgB	-2.3	0E + 00
mdh	-1.8	0E + 00
Cj0539	-2.7	0E + 00

Cj0559	-1.4	0E + 00
Cj0573	-3.4	0E + 00
tatB	-4.8	0E + 00
cbf2	-2.8	0E + 00
ftn	-1.2	0E + 00
hypC	-2.2	0E + 00
Cj0633	-2.5	0E + 00
adk	-1.4	0E + 00
Cj0649	-2.3	0E + 00
hslU	-1.4	0E + 00
rplI	-3.5	0E + 00
rsmH	-1.8	0E + 00
Cj0708	-1.8	0E + 00
rimM	-3.5	0E + 00
Cj0715	-1.7	0E + 00
Cj0716	-1.0	0E + 00
Cj0717	-4.3	0E + 00
Cj0719c	-2.0	0E + 00
Cj0724	-2.1	0E + 00
hisJ	-1.5	0E + 00
grpE	-2.9	0E + 00
dnaK	-1.5	0E + 00
aspB	-1.5	0E + 00
Cj0771c	-1.7	0E + 00
Cj0776c	-2.8	0E + 00
peb2	-1.4	0E + 00
Cj0805	-1.5	0E + 00
Cj0842	-2.0	0E + 00
Cj0854c	-2.8	0E + 00
Cj0892c	-2.6	0E + 00
rpsA	-1.7	0E + 00
pheT	-1.2	0E + 00
Cj0910	-2.3	0E + 00

ciaB	-2.3	0E + 00
cheR	-2.0	0E + 00
Cj0964	-2.3	0E + 00
Cj0993c	-2.8	0E + 00
hemB	-1.4	0E + 00
Cj1000	-6.3	0E + 00
Cj1004	-2.6	0E + 00
Cj1007c	-1.1	0E + 00
aroB	-1.4	0E + 00
Cj1036c	-3.9	0E + 00
Cj1041c	-3.0	0E + 00
Cj1056c	-1.8	0E + 00
Cj1062	-3.5	0E + 00
rpsF	-3.2	0E + 00
ssb	-2.7	0E + 00
bamD	-2.1	0E + 00
fliW	-1.3	0E + 00
Cj1089c	-2.6	0E + 00
yajC	-1.6	0E + 00
tatA	-2.5	0E + 00
gmk	-1.5	0E + 00
tsf	-2.2	0E + 00
Cj1191c	-2.7	0E + 00
Cj1199	-1.6	0E + 00
Cj1200	-2.2	0E + 00
metE	-1.8	0E + 00
metF	-2.6	0E + 00
Cj1219c	-1.9	0E + 00
groS	-1.3	0E + 00
groL	-1.4	0E + 00
dccS	-1.7	0E + 00
Cj1226c	-1.6	0E + 00
htrA	-1.6	0E + 00

hspR	-1.7	0E + 00
glyS	-1.4	0E + 00
Cj1240c	-2.1	0E + 00
hemE	-1.2	0E + 00
Cj1244	-1.6	0E + 00
Cj1247c	-1.8	0E + 00
purD	-1.1	0E + 00
Cj1251	-1.4	0E + 00
racS	-1.4	0E + 00
trmB	-2.3	0E + 00
Cj1289	-1.9	0E + 00
Cj1307	-1.2	0E + 00
mobA	-3.1	0E + 00
Cj1367c	-2.4	0E + 00
Cj1380	-2.9	0E + 00
Cj1381	-3.3	0E + 00
Cj1412c	-3.2	0E + 00
Cj1418c	-1.4	0E + 00
Cj1426c	-1.4	0E + 00
fcl	-1.6	0E + 00
Cj1450	-1.9	0E + 00
Cj1459	-3.1	0E + 00
Cj1465	-1.5	0E + 00
Cj1477c	-1.3	0E + 00
Cj1483c	-3.1	0E + 00
Cj1484c	-2.2	0E + 00
Cj1492c	-1.4	0E + 00
carA	-1.4	0E + 00
Cj1495c	-2.4	0E + 00
Cj1505c	-2.0	0E + 00
Cj1507c	-2.0	0E + 00
Cj1514c	-2.1	0E + 00
Cj1533c	-4.2	0E + 00

dps	-1.5	0E + 00
rloH	-2.2	0E + 00
Cj1564	-1.4	0E + 00
rpsM	-2.6	0E + 00
rpsD	-1.6	0E + 00
rpoA	-2.7	0E + 00
Cj1621	-3.1	0E + 00
Cj1623	-2.5	0E + 00
Cj1626c	-1.3	0E + 00
Cj1637c	-1.8	0E + 00
Cj1640	-2.1	0E + 00
cgpA	-2.0	0E + 00
Cj1680c	-2.5	0E + 00
rplR	-3.1	0E + 00
rplF	-2.5	0E + 00
rplE	-2.8	0E + 00
rplX	-2.3	0E + 00
rpsQ	-3.1	0E + 00
rplP	-2.2	0E + 00
rpsC	-2.8	0E + 00
rplW	-3.1	4E - 16
Cj0055c	-1.2	4E - 16
Cj0261c	-2.5	4E - 16
Cj0510c	-3.0	8E - 16
Cj0834c	-1.3	8E - 16
Cj1021c	-2.5	8E - 16
kpsD	-1.0	1E - 15
cetB	-1.2	1E - 15
rplV	-1.3	2E - 15
cmeR	-1.8	2E - 15
hslV	-1.3	2E - 15
hemN	-2.5	2E - 15
Cj0411	-1.5	2E - 15

Cj0990c	-1.8	7E - 15
Cj0162c	-3.8	7E - 15
ctsD	-1.7	7E - 15
rplD	-1.2	1E - 14
Cj1460	-2.8	1E - 14
rpsK	-1.1	1E - 14
Cj0135	-1.2	1E - 14
hddC	-1.5	2E - 14
nssR	-5.1	3E - 14
Cj0540	-1.6	3E - 14
ddl	-2.0	4E - 14
holA	-2.3	6E - 14
nusB	-3.3	7E - 14
folB	-2.6	1E - 13
Cj0375	-2.2	1E - 13
Cj1417c	-2.7	1E - 13
Cj1496c	-4.0	2E - 13
folK	-1.2	2E - 13
Cj1421c	-1.4	2E - 13
maf3	-1.3	4E - 13
Cj1280c	-1.0	5E - 13
Cj0881c	-4.4	6E - 13
nadD	-1.2	6E - 13
tal	-1.2	7E - 13
Cj1090c	-1.9	7E - 13
selB	-2.5	7E - 13
Cj1497c	-1.5	9E - 13
Cj1642	-1.6	1E - 12
Cj0859c	-1.3	1E - 12
kpsS	-1.1	2E - 12
Cj1415c	-1.8	2E - 12
napL	-2.2	3E - 12
rnpA	-1.2	3E - 12

Cj1631c	-1.1	4E - 12
Cj0044c	-1.1	5E - 12
Cj0519	-2.4	5E - 12
Cj0967	-1.5	6E - 12
flgA	-1.8	8E - 12
Cj0058	-3.0	1E - 11
Cj0600	-1.7	1E - 11
Cj1302	-1.9	1E - 11
Cj0520	-4.5	1E - 11
leuC	-4.5	2E - 11
Cj0331c	-1.2	2E - 11
recN	-1.3	2E - 11
Cj1532	-1.6	2E - 11
Cj1715	-1.2	2E - 11
Cj1436c	-1.1	2E - 11
Cj1172c	-1.3	3E - 11
Cj0898	-1.1	3E - 11
clpS	-2.0	3E - 11
Cj0983	-1.2	3E - 11
pflA	-1.0	3E - 11
Cj0660c	-2.2	3E - 11
ispE	-1.2	4E - 11
Cj1063	-5.6	4E - 11
Cj1340c	-4.1	4E - 11
Cj0067	-1.2	5E - 11
rpmG	-1.8	5E - 11
hddA	-1.1	6E - 11
Cj1349c	-2.0	6E - 11
Cj0190c	-1.1	7E - 11
Cj0253	-2.1	8E - 11
Cj1068	-2.0	8E - 11
Cj1301	-2.7	8E - 11
rpsS	-2.0	1E - 10

Cj0341c	-3.2	1E - 10
Cj0163c	-2.0	1E - 10
rlmH	-1.9	1E - 10
rpmC	-4.0	2E - 10
ispA	-1.5	2E - 10
rpsH	-1.1	2E - 10
Cj0488	-3.4	2E - 10
cysQ	-1.1	2E - 10
fliK	-1.1	3E - 10
parB	-1.1	4E - 10
Cj1374c	-1.2	4E - 10
Cj0124c	-4.7	4E - 10
pyrF	-1.3	5E - 10
Cj0908	-1.4	5E - 10
Cj0682	-1.5	8E - 10
Cj0376	-3.8	1E - 09
Cj0140	-1.4	1E - 09
engB	-1.6	1E - 09
psd	-1.3	1E - 09
acpP2	-1.7	2E - 09
Cj1235	-1.2	2E - 09
Cj1435c	-1.5	4E - 09
Cj1042c	-3.7	4E - 09
Cj0085c	-1.3	5E - 09
csrA	-1.3	6E - 09
Cj1249	-1.2	6E - 09
fliH	-1.2	8E - 09
Cj0033	-1.6	8E - 09
Cj1668c	-2.4	9E - 09
pdxA	-1.1	1E - 08
Cj0151c	-1.6	1E - 08
Cj0515	-1.5	1E - 08
bpt	-1.7	1E - 08

miaA	-1.5	2E - 08
uppS	-1.8	2E - 08
dnaQ	-1.0	2E - 08
Cj0792	-1.8	2E - 08
aat	-1.1	2E - 08
dsbA	-1.1	3E - 08
Cj1115c	-1.1	3E - 08
Cj0963	-2.1	3E - 08
Cj0038c	-3.2	3E - 08
tonB1	-4.1	4E - 08
Cj0653c	-1.1	4E - 08
Cj0139	-1.0	5E - 08
feoA	-3.2	6E - 08
kdtA	-2.8	7E - 08
Cj0422c	-1.6	7E - 08
pglI	-1.4	1E - 07
Cj0111	-1.1	1E - 07
Cj0794	-1.1	1E - 07
queE	-1.1	2E - 07
Cj0608	-1.3	2E - 07
Cj1563c	-1.7	2E - 07
Cj0062c	-1.4	2E - 07
pldA	-1.0	3E - 07
Cj1028c	-2.5	4E - 07
Cj0823	-3.2	5E - 07
Cj1440c	-1.2	6E - 07
Cj0378c	-1.5	7E - 07
hypA	-1.8	7E - 07
Cj1632c	-1.5	1E - 06
Cj0837c	-1.4	2E - 06
rnc	-1.6	2E - 06
secE	-1.9	2E - 06
Cj0119	-2.1	2E - 06

Cj1011	-1.8	2E - 06
moaD	-3.1	2E - 06
Cj1406c	-1.4	3E - 06
Cj0431	-1.3	3E - 06
Cj1136	-1.2	3E - 06
Cj0243c	-1.1	3E - 06
Cj0916c	-1.5	4E - 06
tilS	-1.7	5E - 06
Cj0364	-2.2	5E - 06
Cj0148c	-2.1	5E - 06
Cj1589	-6.5	5E - 06
Cj0844c	-1.6	5E - 06
purS	-6.5	7E - 06
Cj0959c	-1.4	7E - 06
ribA	-1.2	1E - 05
Cj1461	-1.1	1E - 05
tupC	-1.5	2E - 05
Cj0849c	-1.3	2E - 05
Cj0560	-1.1	2E - 05
napD	-1.1	2E - 05
Cj1057c	-1.8	2E - 05
Cj0620	-3.3	3E - 05
Cj1376	-1.3	3E - 05
Cj0090	-3.3	3E - 05
rimP	-1.0	3E - 05
Cj0030	-1.5	3E - 05
leuD	-2.1	3E - 05
Cj1649	-1.7	3E - 05
Cj1138	-1.4	3E - 05
trmL	-1.4	3E - 05
Cj0556	-1.1	3E - 05
Cj1463	-1.1	5E - 05
dprA	-2.2	5E - 05

ctsR	-1.1	6E - 05
cgb	-2.8	9E - 05
Cj0189c	-1.1	1E - 04
Cj1546	-1.0	1E - 04
Cj0120	-2.5	1E - 04
Cj0730	-3.4	1E - 04
Cj0900c	-2.1	1E - 04
Cj1232	-3.4	2E - 04
Cj1162c	-5.4	2E - 04
Cj0267c	-1.1	2E - 04
Cj0808c	-1.4	2E - 04
Cj0302c	-2.1	2E - 04
cmoA	-1.2	3E - 04
Cj0736	-1.1	3E - 04
modC	-1.1	4E - 04
hsdS	-1.9	5E - 04
Cj0829c	-5.0	5E - 04
Cj1254	-2.3	5E - 04
Cj0416	-2.5	6E - 04
Cj0487	-1.0	6E - 04
Cj0584	-1.3	8E - 04
Cj0651	-1.1	1E - 03
Cj0544	-3.5	1E - 03
ktrA	-1.0	1E - 03
bioC	-2.6	1E - 03
Cj1671c	-1.5	2E - 03
aroK	-3.4	2E - 03
Cj0939c	-2.2	2E - 03
Cj0177	-4.5	2E - 03
Cj0864	-1.1	2E - 03
Cj0948c	-2.8	3E - 03
Cj1383c	-4.4	3E - 03
Cj0266c	-1.1	3E - 03

fliR	-1.4	4E - 03
Cj1174	-1.1	5E - 03
Cj0732	-2.5	6E - 03
Cj0988c	-1.9	6E - 03
Cj1583c	-1.5	7E - 03
ctsT	-1.4	8E - 03
Cj0251c	-3.9	9E - 03
Cj1078	-3.8	1E - 02
Cj0728	-2.4	2E - 02
Cj0839c	-2.6	2E - 02
Cj0889c	-1.1	3E - 02
Cj1060c	-2.4	3E - 02
Cj1100	-1.2	3E - 02
Cj0395c	-9.9	4E - 02
Cj0672	-1.2	4E - 02
**Up-regulated**		
Cj0006	2.3	0E + 00
gltB	1.6	0E + 00
ilvD	1.8	0E + 00
Cj0014c	2.4	0E + 00
queC	1.8	0E + 00
nrdA	1.1	0E + 00
Cj0025c	1.5	0E + 00
thyX	1.3	0E + 00
Cj0037c	2.4	0E + 00
flgD	1.3	0E + 00
flgE	1.4	0E + 00
Cj0045c	1.2	0E + 00
fliM	1.3	0E + 00
flhF	1.7	0E + 00
Cj0069	2.7	0E + 00
lctP	1.2	0E + 00
cdtA	1.7	0E + 00

cydA	2.0	0E + 00
Cj0091	2.0	0E + 00
rplU	2.1	0E + 00
rpmA	1.3	0E + 00
obg	1.9	0E + 00
fmt	1.9	0E + 00
atpA	1.4	0E + 00
atpD	1.2	0E + 00
pal	2.0	0E + 00
pfs	1.3	0E + 00
Cj0118	1.2	0E + 00
Cj0123c	2.0	0E + 00
bamA	1.3	0E + 00
trxB	1.5	0E + 00
trxA	2.1	0E + 00
Cj0150c	1.6	0E + 00
ubiA	1.5	0E + 00
Cj0168c	2.0	0E + 00
Cj0172c	2.2	0E + 00
fliI	1.3	0E + 00
dapB	1.8	0E + 00
Cj0203	1.2	0E + 00
Cj0204	1.8	0E + 00
argC	1.8	0E + 00
argD	1.4	0E + 00
secG	1.7	0E + 00
cynT	1.8	0E + 00
rpmI	2.4	0E + 00
pyrC	1.8	0E + 00
zupT	3.0	0E + 00
Cj0264c	2.2	0E + 00
fabZ	2.2	0E + 00
lpxA	1.4	0E + 00

carB	1.2	0E + 00
panB	2.2	0E + 00
fliG	1.5	0E + 00
dxs	2.0	0E + 00
fabH	1.3	0E + 00
rpmF	1.9	0E + 00
fdxA	1.6	0E + 00
ahpC	2.5	0E + 00
flhB	1.5	0E + 00
motA	1.4	0E + 00
Cj0339	2.3	0E + 00
Cj0343c	1.4	0E + 00
fdxB	3.0	0E + 00
Cj0358	1.0	0E + 00
glmM	1.1	0E + 00
Cj0369c	1.5	0E + 00
rpsU	1.5	0E + 00
Cj0385c	2.2	0E + 00
serS	1.3	0E + 00
Cj0390	1.1	0E + 00
Cj0391c	3.0	0E + 00
frdC	1.0	0E + 00
frdA	1.1	0E + 00
frdB	1.3	0E + 00
Cj0412	1.6	0E + 00
Cj0426	1.8	0E + 00
Cj0427	1.5	0E + 00
Cj0428	1.9	0E + 00
mraY	1.6	0E + 00
sdhA	1.7	0E + 00
sdhB	3.1	0E + 00
sdhC	1.9	0E + 00
Cj0461c	2.4	0E + 00

tuf	1.2	0E + 00
rplL	2.3	0E + 00
rpsL	2.9	0E + 00
fusA	1.3	0E + 00
oorA	1.1	0E + 00
oorC	1.1	0E + 00
hemA	2.3	0E + 00
flaG	1.6	0E + 00
Cj0552	1.4	0E + 00
Cj0553	1.4	0E + 00
Cj0555	1.3	0E + 00
ilvI	1.7	0E + 00
Lys-C	1.1	0E + 00
Cj0583	1.7	0E + 00
ligA	2.0	0E + 00
Cj0593c	3.5	0E + 00
Cj0601c	1.7	0E + 00
pstA	3.3	0E + 00
Cj0619	2.1	0E + 00
ilvC	1.3	0E + 00
aspS	1.6	0E + 00
rlpA	1.3	0E + 00
Cj0667	2.5	0E + 00
Cj0669	1.9	0E + 00
dcuB	1.8	0E + 00
uvrB	1.5	0E + 00
ftsA	2.1	0E + 00
flgG	1.4	0E + 00
rplS	1.2	0E + 00
Cj0725c	1.1	0E + 00
cysE	1.8	0E + 00
valS	1.9	0E + 00
tpx	1.4	0E + 00

napB	2.0	0E + 00
flgS	1.8	0E + 00
fliP	1.7	0E + 00
truA	1.9	0E + 00
Cj0832c	1.6	0E + 00
folD	1.5	0E + 00
lepP	1.8	0E + 00
pheS	1.2	0E + 00
Cj0903c	1.8	0E + 00
hup	2.5	0E + 00
Cj0915	1.6	0E + 00
cstA	2.0	0E + 00
prs	1.3	0E + 00
rpiB	1.2	0E + 00
Cj0935c	2.6	0E + 00
atpE	2.8	0E + 00
glnP	3.0	0E + 00
Cj0947c	2.3	0E + 00
cjaB	2.4	0E + 00
cjaA	1.5	0E + 00
Cj1005c	1.2	0E + 00
Cj1006c	1.3	0E + 00
tgt	1.6	0E + 00
livM	1.9	0E + 00
Cj1026c	2.2	0E + 00
gyrA	1.7	0E + 00
lepA	1.3	0E + 00
cmeF	1.3	0E + 00
thiH	1.5	0E + 00
thiG	2.5	0E + 00
moeB	2.1	0E + 00
dapE	2.4	0E + 00
cobB	2.7	0E + 00

thiE	2.1	0E + 00
thiD	2.1	0E + 00
pyrB	1.5	0E + 00
Cj1112c	2.5	0E + 00
pssA	2.0	0E + 00
cheY	1.4	0E + 00
pglJ	1.7	0E + 00
pglK	2.7	0E + 00
waaF	1.4	0E + 00
gmhA1	2.8	0E + 00
hldE	2.4	0E + 00
Cj1153	2.2	0E + 00
rho	2.3	0E + 00
dnaX	1.3	0E + 00
Cj1164c	2.7	0E + 00
Cj1165c	2.0	0E + 00
Cj1166c	2.2	0E + 00
Cj1168c	2.2	0E + 00
Cj1169c	4.5	0E + 00
omp50	2.1	0E + 00
petB	2.4	0E + 00
petA	4.3	0E + 00
mnmG	1.7	0E + 00
dctA	1.1	0E + 00
atpB	2.8	0E + 00
rny	1.2	0E + 00
glcD	1.4	0E + 00
Cj1214c	1.5	0E + 00
kefB	1.4	0E + 00
Cj1241	3.1	0E + 00
Cj1257c	2.4	0E + 00
porA	2.7	0E + 00
hydD	1.4	0E + 00

hydC	3.1	0E + 00
hydB	1.0	0E + 00
hydA	3.9	0E + 00
Cj1270c	1.6	0E + 00
ktrB	2.2	0E + 00
Cj1287c	1.4	0E + 00
accC	1.9	0E + 00
accB	3.2	0E + 00
pseB	2.6	0E + 00
Cj1295	1.5	0E + 00
Cj1306c	1.4	0E + 00
pseA	2.5	0E + 00
Cj1319	2.1	0E + 00
legF	2.5	0E + 00
nrfH	2.4	0E + 00
ruvB	2.4	0E + 00
glmS	1.6	0E + 00
Cj1369	2.0	0E + 00
Cj1375	2.0	0E + 00
Cj1377c	1.5	0E + 00
fldA	2.0	0E + 00
katA	2.0	0E + 00
gapA	1.3	0E + 00
kpsF	1.4	0E + 00
kpsT	2.2	0E + 00
dut	1.4	0E + 00
rimO	1.4	0E + 00
prfB	1.4	0E + 00
truD	1.6	0E + 00
flgI	1.1	0E + 00
Cj1468	1.8	0E + 00
rpsI	1.6	0E + 00
ccoN	1.7	0E + 00

purA	1.6	0E + 00
Cj1500	2.1	0E + 00
putP	1.7	0E + 00
fdhC	1.8	0E + 00
fdhB	1.7	0E + 00
fdhA	1.7	0E + 00
purM	1.8	0E + 00
galU	1.2	0E + 00
acsA	1.3	0E + 00
pxpA	1.5	0E + 00
Cj1548c	2.7	0E + 00
Cj1555c	3.1	0E + 00
nuoN	1.3	0E + 00
nuoL	1.8	0E + 00
nuoK	2.4	0E + 00
nuoJ	2.0	0E + 00
nuoI	2.3	0E + 00
nuoH	1.8	0E + 00
nuoG	1.8	0E + 00
nuoD	2.4	0E + 00
nuoB	2.4	0E + 00
hisF1	1.7	0E + 00
Cj1609	1.6	0E + 00
Cj1613c	1.7	0E + 00
kgtP	1.8	0E + 00
mutY	1.4	0E + 00
sdaC	1.2	0E + 00
Cj1639	2.4	0E + 00
tkt	1.2	0E + 00
Cj1648	1.7	0E + 00
map	1.8	0E + 00
Cj1653c	1.9	0E + 00
nhaA1	1.7	0E + 00

Cj1656c	3.0	0E + 00
p19	2.2	0E + 00
Cj1660	2.1	0E + 00
gltA	2.2	0E + 00
bioB	2.0	0E + 00
rplB	1.1	0E + 00
rlmN	2.0	0E + 00
Cj1725	1.6	0E + 00
flgE2	1.9	0E + 00
Cj0327	1.3	4E - 16
accA	1.0	4E - 16
macB	1.2	4E - 16
napG	1.1	4E - 16
uvrC	1.2	4E - 16
pyrH	1.0	4E - 16
glyQ	1.2	8E - 16
acpP3	1.7	8E - 16
amaA	1.5	8E - 16
fabG	1.1	1E - 15
Cj0310c	1.9	2E - 15
Cj0611c	1.3	3E - 15
dsbI	1.8	5E - 15
lysA	1.1	7E - 15
recG	1.3	8E - 15
Cj1545c	1.1	1E - 14
Cj0125c	2.2	1E - 14
Cj0496	1.2	1E - 14
proA	1.2	2E - 14
ppi	1.3	2E - 14
sstT	1.2	2E - 14
Cj0934c	1.5	3E - 14
hisD	1.0	4E - 14
Cj0178	1.8	5E - 14

tatC	1.4	5E - 14
flhA	1.0	7E - 14
rpmB	1.7	7E - 14
Cj1515c	1.2	7E - 14
lgt	1.1	8E - 14
cfbpB	1.8	2E - 13
aroC	1.1	2E - 13
Cj1361c	1.0	7E - 13
rppH	1.0	8E - 13
livF	1.2	1E - 12
Cj0340	1.0	1E - 12
Cj1038	1.5	1E - 12
Cj1303	1.2	2E - 12
Cj1633	1.2	2E - 12
mnmE	1.2	3E - 12
hisS	1.1	5E - 12
purB	1.0	5E - 12
rpoZ	1.3	5E - 12
purE	1.1	6E - 12
pstC	2.4	6E - 12
modB	1.6	9E - 12
Cj0860	1.4	9E - 12
Cj0353c	1.0	9E - 12
ruvA	1.4	1E - 11
msbA	1.2	1E - 11
Cj1320	1.1	2E - 11
Cj0557c	1.3	2E - 11
Cj0031	1.0	2E - 11
Cj1662	2.0	4E - 11
purL	1.0	4E - 11
Cj1371	1.1	5E - 11
trpB	1.4	5E - 11
mrdB	1.7	6E - 11

Cj1020c	1.1	7E - 11
livG	1.2	1E - 10
Cj0550	1.1	1E - 10
murJ	1.2	2E - 10
Cj1467	1.3	3E - 10
fliQ	1.4	3E - 10
rnhA	1.4	4E - 10
Cj0199c	1.5	9E - 10
nadE	1.1	1E - 09
Cj1513c	1.2	1E - 09
napH	1.1	1E - 09
Cj0848c	1.4	2E - 09
Cj0609c	1.2	2E - 09
tonB3	1.8	3E - 09
Cj1210	1.2	4E - 09
Cj1663	1.3	7E - 09
moaC	1.0	7E - 09
Cj0809c	1.2	8E - 09
ptmA	1.1	8E - 09
cydB	1.6	6E - 08
Cj0571	1.5	1E - 07
Cj1225	1.1	1E - 07
ung	1.1	3E - 07
trpA	1.2	4E - 07
ceuE	1.3	4E - 07
Cj0294	1.1	5E - 07
Cj1255	1.1	8E - 07
exbB2	1.1	1E - 06
thiS	2.1	4E - 06
ubiX	1.0	2E - 05
Cj0035c	3.1	4E - 05
Cj1216c	1.1	1E - 04
Cj0636	1.1	2E - 04

ybeY	1.1	3E - 04
Cj1203c	1.8	4E - 04
Cj0975	1.3	9E - 04
Cj0176c	1.7	3E - 03
Cj0260c	1.4	4E - 03
chuB	1.0	1E - 02

Supplementary Table S2. Measured values of OD_{600} and CFU/mL for *C. jejuni* 81-176 overnight cultures. Cultures were incubated in MH broth in a microaerobic atmosphere (5% O_2, 10% CO_2, 85% N_2) at 42 °C for 24 h.

Sample	OD_{600}	CFU/mL
1	0.101	1.10E+07
2	0.103	1.00E+07
3	0.099	1.20E+07
4	0.101	1.00E+07
5	0.102	1.30E+07

Supplementary Table S3. Common differentially expressed genes and proteins in *C. jejuni* 11168Δ*luxS* compared to *C. jejuni* NCTC 11168.

Gene name	Difference in gene expression	*P*-value	Protein name	Difference in protein expression	*P*-value
rplL	2.26247431	0.00E+00	RplL	6.147657235	9.15E-05
porA	2.740632462	0.00E+00	PorA	2.501590302	3.84E-02
folE	0.643668434	1.04E-04	FolE	2.438784117	2.87E-03
dapB	1.832144943	0.00E+00	DapB	2.252253175	2.59E-04
ctb	0.970918119	6.29E-06	Ctb	2.189983696	1.06E-02
eno	0.760573722	3.47E-10	Eno	2.028894588	3.30E-02
rpoB	0.811037856	1.86E-11	RpoB	2.0190202	7.72E-03
thiG	2.501233072	0.00E+00	ThiG	2.017311265	1.95E-03
purA	1.624247861	0.00E+00	PurA	1.646993538	2.62E-02
ilvC	1.252092764	0.00E+00	IlvC	1.637678504	5.52E-03
guaA	0.329511776	2.56E-02	GuaA	1.601129413	2.06E-02
dapE	2.357275576	0.00E+00	DapE	1.537709634	2.10E-04
leuS	0.462751733	4.34E-04	LeuS	1.496526043	5.29E-03
pseB	2.57038766	0.00E+00	PseB	1.388117433	1.93E-02
aspS	1.633461656	0.00E+00	AspS	1.310820897	2.00E-02
rplT	0.952788717	6.72E-14	RplT	1.297215263	1.09E-02
fabH	1.285473419	0.00E+00	FabH	1.181837161	7.81E-03
dapD	0.296090917	3.04E-02	DapD	1.00121816	4.57E-02
thrS	0.547612082	2.33E-05	ThrS	0.973991315	2.33E-03
hisF1	1.733245891	0.00E+00	HisF1	0.709415674	2.52E-02
cysM	0.353371487	7.33E-03	CysM	0.504441047	3.73E-02
dnaK	-1.488414134	0.00E+00	DnaK	-0.84844477	3.11E-02
recN	-1.282976373	1.79E-11	RecN	-1.096624056	4.57E-02
tupA	-0.296968014	3.37E-02	TupA	-1.195404065	3.58E-02
lpxB	-2.054545385	0.00E+00	LpxB	-1.372042815	7.57E-04
hypA	-1.811222975	6.79E-07	HypA	-1.475221713	2.71E-02
pepA	-0.416597016	4.28E-03	PepA	-1.931097984	1.32E-02

Cj1418c	-1.448429317	0.00E+00	Cj1418c	-2.215515892	1.48E-02
